# Effects of a nutrition intervention on acute and late bowel symptoms and health-related quality of life up to 24 months post radiotherapy in patients with prostate cancer: a multicentre randomised controlled trial

**DOI:** 10.1007/s00520-019-05182-5

**Published:** 2019-11-22

**Authors:** Marina Forslund, Anna Ottenblad, Claes Ginman, Silvia Johansson, Peter Nygren, Birgitta Johansson

**Affiliations:** 1 Department of Immunology, Genetics and Pathology, Uppsala University, Uppsala, Sweden; 2 Department of Nutrition and Dietetics, Karolinska University Hospital, Stockholm, Sweden; 3 Department of Clinical Oncology, Central Hospital, Karlstad, Sweden

**Keywords:** Nutrition intervention, Radiotherapy, Bowel symptoms, Prostate cancer

## Abstract

**Purpose:**

Radiotherapy to the prostate gland and pelvic lymph nodes may cause acute and late bowel symptoms and diminish quality of life. The aim was to study the effects of a nutrition intervention on bowel symptoms and health-related quality of life, compared with standard care.

**Methods:**

Patients were randomised to a nutrition intervention (*n* = 92) aiming to replace insoluble fibres with soluble and reduce intake of lactose, or a standard care group (*n* = 88) who were recommended to maintain their habitual diet. Bowel symptoms, health-related quality of life and intake of fibre and lactose-containing foods were assessed up to 24 months after radiotherapy completion. Multiple linear regression was used to analyse the effects of the nutrition intervention on bowel symptoms during the acute (up to 2 months post radiotherapy) and the late (7 to 24 months post radiotherapy) phase.

**Results:**

Most symptoms and functioning worsened during the acute phase, and improved during the late phase in both the intervention and standard care groups. The nutrition intervention was associated with less blood in stools (*p* = 0.047), flatulence (*p* = 0.014) and increased loss of appetite (*p* = 0.018) during the acute phase, and more bloated abdomen in the late phase (*p* = 0.029). However, these associations were clinically trivial or small.

**Conclusions:**

The effect of the nutrition intervention related to dietary fibre and lactose on bowel symptoms from pelvic RT was small and inconclusive, although some minor and transient improvements were observed. The results do not support routine nutrition intervention of this type to reduce adverse effects from pelvic radiotherapy.

**Electronic supplementary material:**

The online version of this article (10.1007/s00520-019-05182-5) contains supplementary material, which is available to authorized users.

## Introduction

Radiotherapy (RT) is a well-established treatment option for patients with intermediate or high-risk prostate cancer. Despite technical advances in delivery, pelvic RT exposes parts of the bowel to some degree of radiation, and 90% of patients experience a change in bowel habits during treatment [1–3]. Acute symptoms such as diarrhoea, abdominal pain and urgency can occur during the treatment period and may subside after RT completion [1, 4]. In addition, severe acute symptoms increase the risk of late bowel symptoms [5]. Late side effects, i.e. symptoms that persist or develop months to years after RT, can be permanent and progressive in severity and may include diarrhoea, urgency, rectal bleeding and incontinence [6]. Approximately 50% of patients report that their quality of life is affected by late bowel symptoms, and 20–40% report that this effect is moderate or severe [7, 8].

Nutrition interventions (NI) in cancer care can comprise approaches such as dietary counselling and dietary modification [9, 10]. Previous studies have evaluated NI such as elemental diet, fibre supplementation, lactose restriction and modification of fat and fibre intake, in order to reduce bowel symptoms from pelvic RT [2, 9, 11–13]. Dietary fibres can be differentiated into insoluble fibres which increases stool bulk and have a laxative effect, and soluble fibres which are fermented to a higher degree and enhances short-chain fatty acid (SCFA) production [14], which could potentially reduce inflammatory processes [15, 16]. Lactose intolerance may occur from pelvic RT due to a reduction in brush-border enzyme and can contribute to bowel symptoms [17]. A variety of advices on a modified fibre or lactose intake are provided in the clinic to patients undergoing pelvic RT, which reflects the lack of consensus in this area [18]. Previous NI have shown some benefits in reducing bowel symptoms from pelvic RT, but there is still not enough evidence, and there is a need for high-quality studies with long-term follow-up [2, 9, 12, 13].

We have previously conducted a randomised controlled trial (RCT), with an NI aiming for a reduced intake of insoluble fibre and lactose, among men with localised prostate cancer undergoing curative RT restricted to the prostate gland. Descriptive data revealed a tendency towards less acute bowel symptoms in the intervention group. This trend did not persist in the long-term evaluation [11, 12]. However, larger irradiated volumes, including the prostate gland and pelvic lymph nodes, may increase bowel symptoms and thereby the benefit from the NI [19].

### Aim

The aim of the paper is to study the effects of an NI, aiming to replace foods high in insoluble fibre and lactose with foods with a higher proportion of soluble fibre and low in lactose, on acute and late bowel symptoms and health-related quality of life (HRQoL), among men undergoing RT to the prostate gland and pelvic lymph nodes, compared with standard care.

## Material and methods

### Design

This study is a RCT evaluating the same NI on the same outcome measures as in our previous RCT [11, 12], i.e. bowel symptoms as the primary outcome measure and HRQoL as the secondary outcome measure.

### Patients

**Fig. 1:**
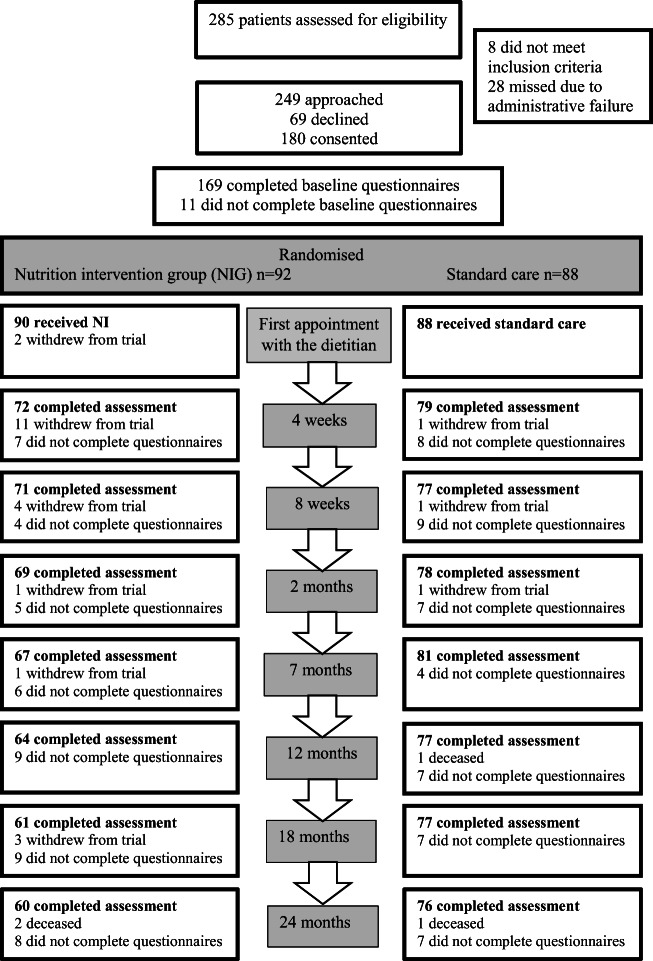
Flow chart. Note: ‘Did not complete questionnaires’: the number of patients who did not complete the specific assessment point but did not withdraw from the study. First appointment with the dietitian: the start of radiotherapy, 4 and 8 weeks: after the start of radiotherapy, 2, 7, 12, 18 and 24 months: after radiotherapy completion

From October 2009 to January 2014, consecutive patients with intermediate- or high-risk localised prostate cancer referred to curative RT, at Uppsala, Karlstad or Gävle hospital in Sweden, were assessed for eligibility. Exclusion criteria were cognitive impairment, previous RT to the pelvis, inflammatory bowel disease, need for long-term hospital care or inability to speak or understand Swedish. Eligible patients received written and oral information about the study during a visit to the hospital or by telephone. One hundred and eighty (72%) of 249 approached patients gave their informed consent to participate (Fig. 1). The Regional Ethical Review Board in Uppsala approved the study (Dnr 2009/209).

### Radiotherapy

Patients treated in Uppsala received irradiation of the prostate, seminal vesicles and pelvic lymph nodes with intensity-modulated radiation technique (IMRT) or volumetric modulated arc therapy (VMAT) (Table 1). A prostate boost was given with brachytherapy, with a fortnight’s pause halfway through the IMRT/VMAT treatment, or by protons, or photons using 3D-conformal EBRT, with a 1-week pause before the IMRT/VMAT treatment. Patients in Karlstad received irradiation of the prostate, seminal vesicles and pelvic lymph nodes with IMRT, and a boost to the prostate. Patients in Gävle received irradiation of the prostate, seminal vesicles and pelvic lymph nodes with rapid arc technique, and a boost to the prostate.

**Table 1:** Radiotherapy techniques used in the treatments of patients with intermediate- or high-risk prostate cancer at three oncology departments

	Number of patients	Clinical target volume	Fraction size (Gy)	Dose (Gy)	Total dose (Gy)	Overall treatment time (weeks)
Uppsala						
IMRT/VMAT with brachytherapy boost	54	Prostate gland, seminal vesicles and pelvic lymph nodes; prostate gland	2 × 25; 10 × 2	50; 20	70	7
IMRT/VMAT with proton/photon boost ^a^	49	Prostate gland, seminal vesicles and pelvic lymph nodes; prostate gland	2 × 25; 5 × 4	50; 20	70	7
IMRT/VMAT with boost	4	Prostate gland, seminal vesicles and pelvic lymph nodes; prostate gland	2 × 25; 2 × 10	50; 20	70	7
Karlstad						
IMRT with boost^b^	35	Prostate gland, seminal vesicles and pelvic lymph nodes; prostate gland	2 × 25; 2 × 8 + 2 × 7 ^a^	50; 30	80	8
Gävle						
Rapid Arc IMRT with rapid arc boost	38	Prostate gland, seminal vesicles and pelvic lymph nodes; prostate gland	2 × 25; 2 × 14	50; 28	78	8

*IMRT* intensity-modulated radiation technique, *VMAT* volumetric modulated arc therapy

^a^To reduce the dose to the rectal wall, a rectal retraction rod was fixed to a length of 7–8 cm and was in place at the CT for treatment planning and during each proton or photon boost fraction

^b^Boost to the prostate, delivered with IMRT technique 2 × 8 Gy and conventional technique 2 × 7 Gy, to a total of 30 Gy

### Power analysis and randomisation

A 5-point change in the European Organisation for Research and Treatment of Cancer quality-of-life questionnaire (EORTC QLQ-C30) scales is considered clinically significant [20]. Given a mean difference of five in bowel symptoms between groups, and using a standard deviation of 9.4 from previous research [21], the effect size was calculated to 0.53. To reach a power of 80%, with our estimated effect size and a 0.05 significance level, 57 patients in each group were required. Expecting an attrition rate of approximately 30% due to the extensive follow-up period, we decided to include 90 patients in each group. Patients were stratified by radiation technique and site, and randomly assigned to a nutrition intervention group (NIG, *n* = 92) or a standard care group (SCG, *n* = 88). Randomisation was performed by two persons unrelated to the trial, using the Efron’s biased coin design [22].

### The nutrition intervention

The NI comprised three individual sessions (approximately 30–60 min) with a research dietitian, face-to-face at baseline (onset of RT) and 4 weeks after baseline (mid treatment), and by telephone at 8 weeks (end of treatment). Spouses were welcome to participate. The NIG was advised to replace foods high in insoluble fibre and lactose with foods with a high proportion of soluble fibre and low in lactose, during the entire study period (26 months). The dietary advice was standardised and food items were categorised as ‘recommended’ and ‘not recommended’ (Supplementary File A). Advice regarding boiling of vegetables and mixing of soups to aid digestion was also provided. A dietary advice pamphlet was handed out at baseline and sent by mail as a reminder at all assessment points except for the last at 26 months. The NIG completed food records at two occasions, according to the same purpose and methods described previously [11].

### Standard care

The SCG was recommended to continue their habitual diet. Routine dietary counselling was not part of the standard care for this patient category, but dietitian consultation was offered if needed. Two patients in the SCG received dietary counselling regarding bowel symptoms and one about nutritional drinks.

### Data collection

Bowel symptoms, HRQoL and dietary adherence were assessed at eight assessment points (Fig. 1). All patients completed baseline data collection before being informed about the randomisation.

### Clinical and demographic characteristics

Demographics and medical data were collected from medical records or obtained at the baseline visit. Information regarding proctitis, eventual lower intestinal endoscopies, urinary tract infection, use of antibiotics and hospitalisation during and after RT were collected from medical records at 1 and 24 months after RT completion. Information regarding cancer recurrence and additional oncological treatment were collected at 24 months. Nutritional status was assessed with the Scored Patient-Generated Subjective Global Assessment (PG-SGA) tool at baseline and the PG-SGA Short Form at 12 and 24 months after RT [23, 24].

### Bowel symptoms and HRQoL

Diarrhoea and constipation were assessed with the EORTC QLQ-C30 [25]. Limitations to daily activities due to bowel symptoms, unintentional leakage of stools, blood in stools and bloated abdomen were assessed with the prostate-specific module QLQ-PR25 [21]. Patient-perceived bother from eight bowel symptoms was assessed with the Gastrointestinal Side Effects Questionnaire (GISEQ). Two additional questions asked for other bowel symptoms (yes/no) and use of medication due to bowel symptoms (Yes/No) [11, 12, 26]. Additional aspects of HRQoL, including global health status, functioning and symptoms, were assessed with EORTC QLQ-C30 and QLQ-PR25.

### Dietary adherence

A food frequency questionnaire (FFQ) concerning the intake of 61 fibre- and lactose-containing food items during the previous month (never/less than once a month up to ≥ 3 times/day) was completed at all assessments. Two additional questions concerned use of lactose-reduced dairy products (yes/no) and type of products. No portion sizes were measured.

FFQ data were calculated into adherence scores, an approach used in previous studies [27–29]. Food items with a median of ≥ 2 times/month (*n* = 30) were categorised into not recommended and recommended grain products, not recommended and recommended vegetables and high-lactose and low-lactose dairy products. The median intake per day for each of the six food categories was calculated for all assessment points (Supplementary File, B). The food categories were dichotomised using the group median and assigned a value of 0 (not recommended) or 1 (recommended foods). Use of lactose-free/reduced dairy products was scored 1 (yes) or 0 (no). In this way, each patient was assigned an adherence score at each assessment point ranging from 0 (low adherence) to 7 (high adherence).

### Statistical analyses

Analyses were performed using IBM SPSS Statistics version 24.0 and R version 3.4.3 (multivariate linear regression, MLR, analyses). The QLQ-C30 and QLQ-PR25 were scored in accordance with the EORTC scoring manual [30]. Bowel symptoms in the QLQ-PR25 were analysed both as single items and as the bowel symptoms scale [21]. MLR was used to analyse how the NI was associated with bowel symptoms and HRQoL during the acute phase (the overall mean value of the assessment at 4 weeks and 8 weeks after RT start, and 2 months post-RT) and the late phase (the overall mean value of the assessment at 7, 12, 18 and 24 months post-RT). All models were adjusted for the baseline value, age at randomisation, radiotherapy dose, diabetes and smoking. Models analysing the late phase were also adjusted for acute phase value.

Analyses were conducted on an intention-to-treat (ITT) and per protocol [31]. Missing data during the acute or late phase in the ITT analysis were substituted with the mean of the patient’s responses [32], provided that baseline and at least one assessment during the acute or late phase had been completed. Patients in the NIG who were considered adherent (*n* = 27), i.e. an adherence score ≥ 3 at all assessment points during the acute phase, were included in the per-protocol analyses and compared with the patients in the SCG who did not have a reduced intake of insoluble fibre and lactose (i.e. an adherence score < 3 during the acute phase, (*n* = 47)) [31]. The number and proportion of patients reporting bowel symptoms to be at least ‘a little’ and ‘quite a bit’ bothersome, at one or more assessments, were calculated for QLQ-C30 and QLQ-PR25 single-item symptoms. The Student’s unpaired *t* test and the chi-square test were used to analyse differences between groups at baseline. Charlson’s Comorbidity Index was used to calculate the comorbidity burden [33].

## Results

No statistically significant differences between the NIG and the SCG were found regarding baseline characteristics (Table 2), bowel symptoms at baseline, proctitis, urinary tract infection, use of antibiotics, hospitalisation, additional oncological treatment or cancer recurrence, at 1 and 24 months after RT completion. Eight of the 151 patients who completed their participation in the trial had cancer recurrence documented in the medical journals at 24 months after RT. Six patients in the NIG and two in the SCG received adjuvant chemotherapy (docetaxel) after RT completion, and one patient in the NIG and one in the SCG underwent chemotherapy (docetaxel) due to cancer recurrence.

**Table 2:** Demographic and clinical characteristics among patients with prostate cancer undergoing radiotherapy, who received the nutrition intervention (NIG) and those who received standard care (SCG)

	NIG, *n* = 92^a^	SCG, *n* = 88
Age (years)		
Mean (SD)	67.3 (5.3)	67.1 (5.5)
PSA (ng/ml)		
Mean (SD)	27.3 (38.7)	25.3 (31.3)
Clinical stage (*n*)		
T1	7	7
T2	17	19
T2–T3	7	7
T3	52	51
T4	5	2
Not available	3	2
Gleason score (*n*)		
6	3	8
7	25	26
8	29	23
9	28	25
10	4	5
Not available	2	1
Treatment modality (*n*)		
IMRT	40	37
IMRT + brachytherapy boost	28	26
IMRT + proton/photon boost	24	25
Endocrine therapy (*n*)		
Yes	87	84
No	4	4
Charlson Comorbidity Index		
Mean (SD)	1.0 (1.1)	1.0 (1.2)
Diabetes (*n*)		
Yes	13	14
No	78	74
Treated with anticoagulants (*n*)		
Yes	24	24
No	67	64
Height (cm)		
Mean (SD)	177.7 (6.2)	178.3 (5.7)
Weight (kg)		
Mean (SD)	86.8 (14.2)	88.1 (13.3)
BMI (*n*)		
Underweight	0	1
Normal	29	21
Overweight	38	45
Obese	24	21
Scored PG-SGA total score		
Mean (SD)	2.5 (1.8)	2.2 (1.1)
Scored PG-SGA global rating (*n*)		
A: Well-nourished	88	86
B: Moderately malnourished	3	2
Marital status (*n*)		
Married/cohabitant	76	64
Single/divorcee	11	17
Unknown	4	7
Smoking (*n*)		
Current smoker	8	11
Never smoked	36	34
Former smoker	43	36
Unknown	4	7

*BMI* body mass index WHO definition, *IMRT* intensity-modulated radiotherapy, *PG-SGA* the scored Patient-Generated Subjective Global Assessment tool

^a^One patient did not complete dietitian baseline assessment; thus, data are not available, Charlson Comorbidity Index score ranges from 0 to 37

### Dietary adherence

The most obvious changes were observed during the acute phase. The patients in the NIG reduced their intake of non-recommended grains, bread, vegetables and fruits and increased their intake of recommended grains, bread, vegetables and fruits (Supplementary File, B). Adherence subsided during the late phase. The intake of dairy products was stable throughout the study period; the use of or lactose-reduced products was most frequent during the acute phase.

### NI associations with bowel symptoms

**Fig. 2:**
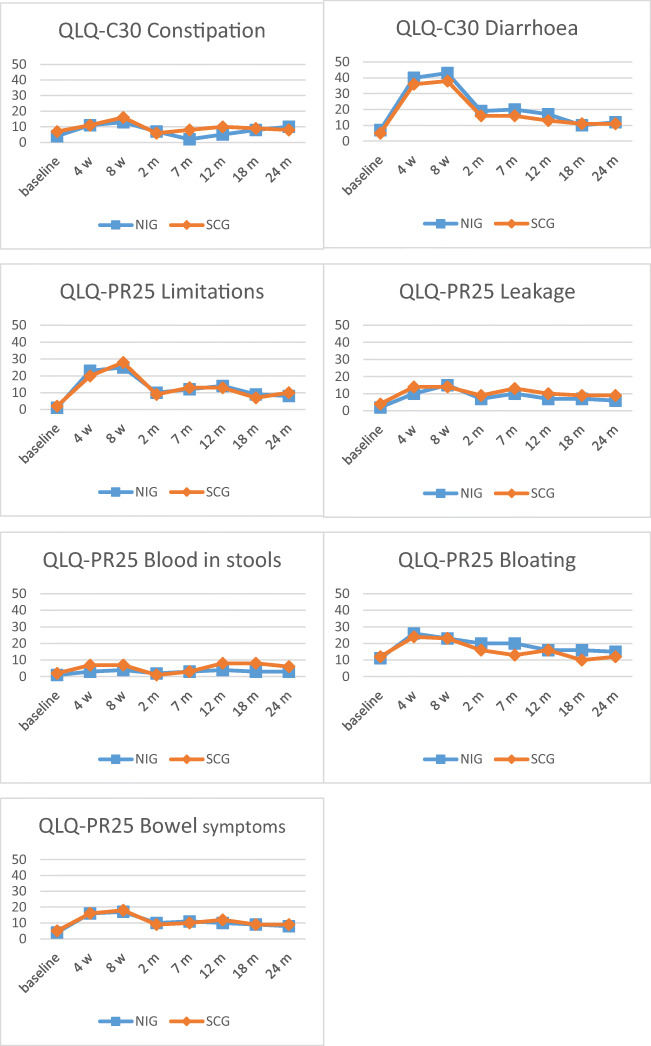

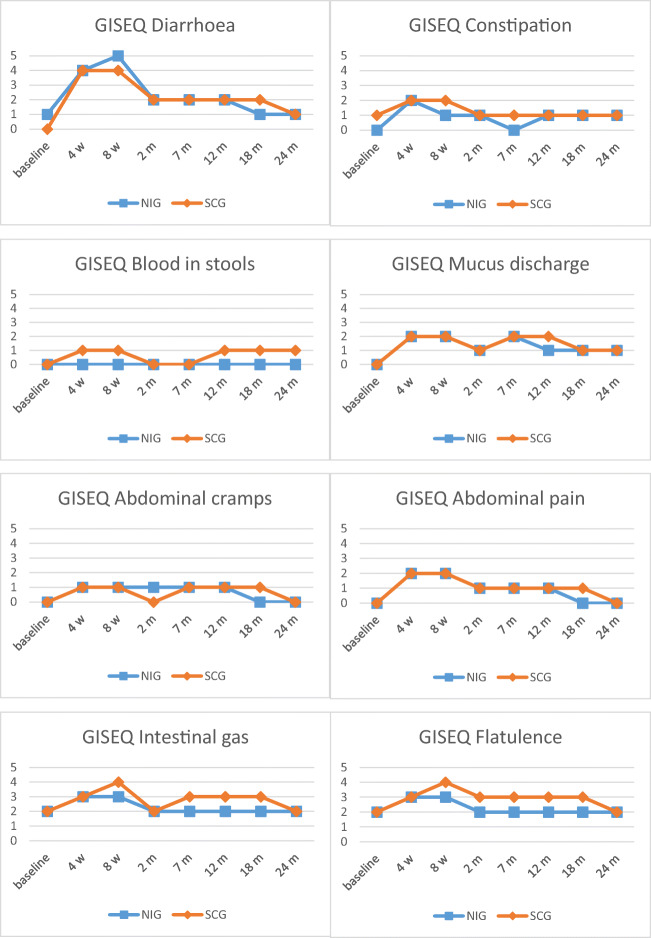
Mean scores for bowel symptoms at baseline, 4 weeks, 8 weeks, 2 months, 7 months, 12 months, 18 months and 24 months. Variables from the QLQ-C30, QLQ-PR25 and GISEQ assessing bowel symptoms among patients with prostate cancer undergoing radiotherapy, who received the nutrition intervention (NIG) and those who received standard care (SCG). Abbreviations: Bloating, bloated abdomen; Bowel symptoms, aggregated scale bowel symptoms; Limitations, limitations of daily activities due to bowel symptoms; Leakage, unintentional leakage of stools. Note: Scores ranges from 0 to 100 in EORTC QLQ-C30 and QLQ-PR25, and from 0 to 10 in GISEQ

The baseline levels of bowel symptoms were low in both groups. Most bowel symptoms worsened from baseline during the acute phase and then improved during the late phase, although not returning completely to baseline levels (Fig. 2, Table 3). The NI was associated with less bother from blood in stools (*p* = 0.047) and less bother from flatulence (*p* = 0.014) during the acute phase but these differences were not clinically significant. However, the NI was associated with an increase in bloated abdomen during the late phase (*p* = 0.029) (Table 3). There were no associations between the NI and bowel symptoms in the per-protocol analyses (data not shown).

**Table 3:** Mean scores (SD) for variables assessing bowel symptoms at baseline, the acute phase and the late phase, among patients with prostate cancer undergoing radiotherapy, who received a nutrition intervention (NIG) and those who received standard care (SCG)

	Baseline, mean (SD)		Acute phase^1^, mean (SD)		Late phase^2^, mean (SD)	
	NIG	SCG	NIG	SCG	NIG	SCG
	*n* = 84	*n* = 85	*n* = 75	*n* = 82	*n* = 68	*n* = 82
EORTC QLQ-C30						
Diarrhoea	7 (15)	5 (13)	35 (22)	31 (21)	15 (19)	13 (19)
Constipation	4 (12)	7 (17)	10 (16)	12 (17)	6 (12)	9 (19)
EORTC QLQ-PR25						
Limitations	1 (5)	2 (9)	20 (22)	20 (18)	11 (19)	11 (16)
Leakage	2 (9)	4 (12)	10 (14)	13 (17)	7 (12)	10 (17)
Blood	1 (5)	2 (10)	3 (8)	5 (11)	3 (7)	7 (13)
Bloating	11 (20)	12 (20)	22 (23)	22 (22)	18 (21)*	13 (19)
Bowel symptoms	4 (7)	5 (8)	14 (11)	15 (12)	10 (10)	10 (13)
GISEQ						
Diarrhoea	1 (1)	0 (1)	4 (2)	3 (3)	2 (2)	2 (2)
Constipation	0 (1)	1 (2)	1 (2)	1 (2)	1 (1)	1 (2)
Blood	0 (0)	0 (1)	0 (1)*	1 (1)	0 (1)	1 (2)
Mucus	0 (1)	0 (1)	2 (2)	2 (2)	1 (2)	2 (2)
Cramps	0 (0)	0 (0)	1 (1)	1 (2)	1 (1)	1 (1)
Pain	0 (1)	0 (1)	1 (2)	2 (2)	1 (1)	1 (2)
Intestinal gas	2 (2)	2 (2)	3 (2)	3 (2)	2 (2)	3 (3)
Flatulence	2 (2)	2 (2)	2 (2)*	3 (2)	2 (2)	3 (2)

*Blood* blood in stools, *Bloating* bloated abdomen, *Bowel symptoms* aggregated scale bowel symptoms, *Cramps* abdominal cramps, *Limitations* limitations of daily activities due to bowel symptoms, *Leakage* unintentional leakage of stools, *Mucus* mucus discharge, *Pain* abdominal pain. Scores range from 0 to 100 in EORTC QLQ-C30 and QLQ-PR25, and from 0 to 10 in GISEQ

^1^Acute phase: 4 weeks, 8 weeks and 2 months

^2^Late phase: 7, 12, 18 and 24 months

*Significant associations between the NI and more bloated abdomen *p* = 0.029, less bother from blood in stools *p* = 0.047 and less bother from flatulence *p* = 0.014

### Covariate associations with bowel symptoms

Higher levels of bowel symptoms at baseline were associated with higher levels of symptoms during the acute phase (*p* < 0.004), except for constipation, blood in stools, abdominal cramps and limitations to daily activities due to bowel symptoms. Higher levels of constipation, abdominal cramps, unintentional leakage of stools, bloated abdomen and bowel symptoms at baseline were associated with more symptoms during the late phase (*p* < 0.001). More bowel symptoms during the acute phase were associated with more symptoms during the late phase (*p* < 0.0001–0.043), except for blood in stools. Higher radiation dose was associated with more constipation during the acute phase (*p* = 0.030). Former smoking was associated with a less bloated abdomen during the acute phase (*p* = 0.037), using never smokers as a reference.

### Prevalence of bowel symptoms

Diarrhoea was the most prevalent symptom during the acute phase, 76% in the NIG and 69% in the SCG reported at least ‘quite a bit’ of diarrhoea (Table 4). Other symptoms rated ‘quite a bit’ during the acute phase were limitations to daily activities due to bowel symptoms and bloated abdomen. Bloated abdomen was also the most common symptom during the late phase. Blood in stools was less prevalent in the NIG compared with the SCG during the acute and the late phase. There were no differences between the groups regarding the self-reported data on other bowel symptoms, or use of medication due to bowel symptoms during or after RT (data not shown).

**Table 4:** Data from the EORTC QLQ-C30 and QLQ-PR25. Patients with prostate cancer undergoing radiotherapy who reported any level of symptoms (≥ 33) and ‘quite a bit’ or ‘very much’ symptoms (≥ 67) at one or more occasions during the acute or late phase, among patients who received a nutrition intervention (NIG) and those who received standard care (SCG)

	Baseline				Acute phase^1^				Late phase^2^			
	NIG: *n* = 84, SCG: *n* = 85				NIG: *n* = 75, SCG: *n* = 82				NIG: *n* = 68, SCG: *n* = 82			
EORTC transformed scores 0-100	≥ 33		≥ 67		≥ 33		≥ 67		≥ 33		≥ 67	
	NIG, *n* (%)	SCG, *n* (%)	NIG, *n* (%)	SCG, *n* (%)	NIG, *n* (%)	SCG, *n* (%)	NIG, *n* (%)	SCG, *n* (%)	NIG, *n* (%)	SCG, *n* (%)	NIG, *n* (%)	SCG, *n* (%)
EORTC, QLQ-C30												
Diarrhoea	15 (18)	13 (15)	2 (2)	1 (1)	69 (92)	72 (88)	57 (76)	57 (69)	42 (62)	43 (52)	24 (35)	26 (32)
Constipation	8 (9)	15 (18)	1 (1)	4 (5)	31 (41)	42 (51)	18 (24)	23 (28)	19 (28)	27 (33)	12 (18)	17 (21)
EORTC, QLQ-PR25												
Limitations	2 (2)	6 (7)	0 (0)	0 (0)	47 (63)	56 (69)	29 (39)	38 (47)	28 (41)	38 (47)	16 (24)	26 (32)
Leakage	6 (7)	9 (11)	0 (0)	1 (1)	33 (45)	41 (51)	21 (28)	27 (33)	27 (41)	36 (44)	12 (18)	21 (26)
Blood	2 (2)	5 (6)	0 (0)	1 (1)	13 (18)	18 (23)	4 (6)	11 (14)	14 (21)	23 (28)	7 (10)	13 (16)
Bloating	23 (27)	25 (30)	4 (5)	4 (5)	52 (69)	53 (65)	35 (47)	42 (52)	42 (63)	41 (51)	29 (43)	29 (36)

*Blood* blood in stools, *Bloating* bloated abdomen, *Limitations* limitations of daily activities due to bowel symptoms, *Leakage* unintentional leakage of stools

^1^Acute phase: 4 weeks, 8 weeks and 2 months

^2^Late phase: 7, 12, 18 and 24 months

### NI associations with HRQoL

Global health status, functioning and symptoms worsened during the acute phase and improved during the late phase, although not returning to baseline levels for all variables, and without obvious differences between the groups (Supplementary File, C). Dyspnoea worsened during the late phase. Urinary symptoms were the worst symptoms during the acute phase, and fatigue, insomnia and hormonal treatment–related symptoms during the late phase. The NI was associated with more loss of appetite during the acute phase (*p* = 0.018). No other associations were found between the NI and HRQoL domains.

### Covariate associations with HRQoL

Higher functioning and more symptoms at baseline were associated with higher functioning and more symptoms during the acute and late phases (*p* < 0.0001–0.027). Exceptions were sexual functioning during the acute phase, and global health status, cognitive functioning, nausea and vomiting, pain, dyspnoea and sexual functioning during the late phase. A higher acute phase value was associated with higher functioning and more symptoms during the late phase (*p* < 0.001), except for appetite loss and sexual functioning. There were some scattered associations between radiation dose, age, diabetes, smoking and HRQoL domains, but no obvious pattern or strong associations were found (data not shown).

## Discussion

The observed associations between the NI and bowel symptoms were small and inconclusive. The NIG reported statistically, but not clinically significant, less bother from blood in stools and flatulence in the acute phase, but more bloated abdomen during the late phase. Also, the NI seemed to affect the patients’ appetites negatively during the acute phase. Thus, the results do not provide support for an NI aiming to replace foods high in insoluble fibre and lactose with foods with a higher proportion of soluble fibre and low in lactose, during RT against prostate cancer. Quite a large proportion of the NIG were not considered adherent, making it difficult to draw firm conclusions regarding the effects of the NI. The lack of strong effects from the NI is supported by the per-protocol analyses, since no differences were found between adherers and patients who did not have a reduced intake of insoluble fibre and lactose.

Furthermore, it must be taken into consideration that the distinction between soluble and insoluble dietary fibres is complex [34]. A recent RCT found that a high-fibre diet–reduced gastrointestinal toxicity compared with habitual fibre intake, both in the acute and late phases [35]. A higher fibre intake will also increase the intake of soluble fibres which may be beneficial due to the enhanced production of SCFA, which could potentially reduce inflammatory processes [16]. Further investigations of the effects of a high-fibre diet during pelvic RT are recommended.

Blood in stools and flatulence may be induced by pelvic RT [8], and the associations between the NI and these symptoms may indicate some small effects of the NI. However, the difference between the groups was minor, and of small or no clinical significance. The NI was associated to more loss of appetite during the acute phase; this may be explained by the reason that the NIG had to change their diet during pelvic RT which may also cause appetite loss [36]. The NIG experienced more bloated abdomen during the late phase compared with the SCG; the five-point difference was small but may be considered clinically significant [20]. This difference may be due to an increasing intake of fibre and lactose over time. Production of gas is a known side effect of fibre ingestion and unabsorbed lactose and can cause discomfort and bloating [37, 38].

The pattern of worsening bowel symptoms during the acute phase fits well with previous research [1, 6]. More than one of four patients were bothered ‘quite a bit’ of diarrhoea, bloated abdomen and/or limitations to daily activities due to bowel symptoms during the late phase. This corroborate earlier findings [7, 8] and highlights the need for interventions to decrease late bowel symptoms from extended field RT for prostate cancer. Our previous RCT revealed a similar pattern of acute and late bowel symptoms [11, 12]; however, a larger proportion of patients in the present study reported ‘quite a bit’ of late bowel symptoms, indicating that a larger irradiated volume, as expected, increased bowel symptoms and decreased HRQoL. Ten patients started chemotherapy during the late phase. Side effects from chemotherapy may have contributed to some of the late symptoms, and this should be taken into consideration when interpreting the results.

The importance of screening for pre-existing bowel symptoms in order to adequately evaluate radiotherapy-induced bowel symptoms has been highlighted [39]. We observed that higher levels of bowel symptoms at baseline were associated with more symptoms during both the acute and the late phases for the majority of symptoms. Furthermore, more bowel symptoms during the acute phase were associated with more bowel symptoms during the late phase except for blood in stools. All patients in the NIG received the same dietary advice, whether they had pre-existing symptoms or not. It is possible that screening for pre-existing bowel symptoms before RT, and targeting tailored NI to patients with symptoms, could be beneficial.

HRQoL is generally high among patients with prostate cancer treated with RT and comparable with normative data, but symptoms such as bowel and urinary problems and sleep disturbances are more pronounced [40, 41]. In our study, there were clinically significant impairments in functioning and symptoms [20] during the acute phase which did not recover completely during the follow-up period, again, pointing to the need for supportive interventions for patients with prostate cancer undergoing RT.

### Methodological discussion

A strength of this study is its experimental design with long-term follow-up until 2 years after RT completion. However, NI studies requiring long-term adherence are not without difficulties. Twenty-two (24%) patients in the NIG, and three in the SCG, withdrew from the study, the majority of them during the acute phase. Since withdrawal was not balanced between groups, it is probably not random and is thus difficult to adjust for and might decrease the internal validity of the study. Dietary counselling was conducted at three times during the acute phase. Adherence could possibly have been improved if there had been more dietitian appointments, during both the acute and the late phases, as suggested by the patients in a qualitative interview study [42]. Furthermore, many patients had to travel quite a distance to their treatments, which might have affected their perseverance in planning and preparing food according to the dietary advice.

Another limitation is that the reduction of insoluble fibre and lactose might have been too small to be effective. No target levels for the insoluble fibre and lactose reduction were defined, and it is possible that defined goals for reduction in insoluble fibre and lactose, and an index for self-assessment of adherence, would have been helpful. On the other hand, this would impose a greater workload on the patients. Finally, another limitation is that an extended follow-up period (> 5 years) is required to fully appreciate late bowel symptoms, since such symptoms may worsen beyond 2 years [43].

To conclude, the effect of a modified intake of dietary fibre and lactose on bowel symptoms from pelvic RT was small and inconclusive, although some minor and transient improvements were observed. The results do not support routine NI of this type to reduce adverse effects from pelvic RT. There is a need for more NI studies to reduce pelvic radiotherapy-induced bowel symptoms.

## Electronic supplementary material

ESM 1 (DOCX 24 kb)
