# Supplementary material for: Effects of a nutrition intervention on acute and late bowel symptoms and health-related quality of life up to 24 months post radiotherapy in patients with prostate cancer: a multicentre randomised controlled trial
Source: Support Care Cancer. 2019 Nov 22;28(7):3331–42. doi: 10.1007/s00520-019-05182-5 (PMC7256032; doi:10.1007/s00520-019-05182-5)
Supplement: Supplementary file 1 — (DOCX 24 kb) [file 520_2019_5182_MOESM1_ESM.docx]

**Supplementary file A**. Examples of the dietary advice in the nutrition intervention among patients with prostate cancer undergoing radiotherapy.

| **Food group** | **Foods recommended** | **Foods not recommended** |
| --- | --- | --- |
| Vegetables | Tender vegetables, cooked or canned | Raw vegetables with tough skin or seeds. Leguminous plants |
| Fruits and berries | Canned or tender fruit, peeled fruits | Fruit with tough skins, prune juice |
| Potatoes, pasta and rice | Peeled potatoes, white pasta or rice | Potatoes with skins, whole-grain pasta and rice |
| Soups | Soups with smooth texture | Soups with peas, beans or cabbage |
| Bread and biscuits | Bread with wheat, oats, or sifted rye flour | Bread with whole-grain flours, bran or seeds |
| Cereals and grains | Cereals from maize, wheat or oats | Whole grain cereals |
| Dairy products | Lactose-free or lactose-reduced products | High-lactose products |

|  | Baseline  Median  (min-max)  NIG n=90  SCG n=88 | 4 weeks  Median  (min-max)  NIG n=72  SCG n=78 | 8 weeks  Median  (min-max)  NIG n=71  SCG n=77 | 2 months  Median  (min-max)  NIG n=69  SCG n=78 | 7 months  Median  (min-max)  NIG n=67  SCG n=81 | 12 months  Median  (min-max)  NIG n=64  SCG n=77 | 18 months  Median  (min-max)  NIG n=60  SCG n=77 | 24 months  Median  (min-max)  NIG n=60  SCG n=77 |
| --- | --- | --- | --- | --- | --- | --- | --- | --- |
| Grain products not recommended  NIG  SCG | 2.1 (0-7.0)  2.0 (0.1-7.0) | 0.2 (0-4.5)  2.0 (0-5.0) | 0.1 (0-4.0)  1.4 (0-5.0) | 0.6 (0-4.1)  1.3 (0-7.0) | 1.0 (0-5.0)  1.4 (0.1-8.0) | 1.0 (0-5.0)  1.6 (0.1-8.0) | 1.0 (0-4.0)  1.3 (0.1-6.0) | 1.1 (0-5.0)  1.3 (0-4.0) |
| Recommended grain products  NIG  SCG | 1.7 (0-8.8)  1.5 (0-7.1) | 2.4 (0.1-8.7)  1.9 (0.1-6.9) | 2.5 (0-10.3)  1.6 (0-8.4) | 2.4 (0-11.6)  1.5 (0.1-10.0) | 2.3 (0-8.3)  1.6 (0.1-9.6) | 1.9 (0-9.6)  1.5 (0-6.4) | 2.0 (0-7.3)  1.4 (0.1-5.7) | 2.2 (0-7.0)  1.6 (0.1-8.1) |
| Vegetables not recommended  NIG  SCG | 1.8 (0-8.0)  2.4 (0.1-6.4) | 1.0 (0-3.4)  2.0 (0.1-6.3) | 1.0 (0-5.0)  1.8 (0.1-9.3) | 0,7 (0-7.3)  1.7 (0.3-9.0) | 1.2 (0.1-5.3)  1.7 (0.2-8.1) | 1.4 (0-11.1)  1.9 (0-8.0) | 1.4 (0.1-5.2)  1.7 (0.1-9.0) | 1.6 (0.2-6.0)  1.9 (0.1-7.3) |
| Recommended vegetables  NIG  SCG | 1.7 (0.1-6.1)  1.7 (0.4-10.0) | 2.1 (0.2-6.4)  2.0 (0.3-10.3) | 1.9 (0.1-8.0)  1.9 (0.3-12.0) | 1.9 (0.4-5.6)  1.7 (0.3-14.0) | 1.7 (0.4-7.0)  1.7 (0.4-6.0) | 2.1 (0.4-9.3)  1.8 (0.6-6.0) | 1.7 (0.2-5.3)  1.9 (0.4-7.6) | 1.6 (0.2-7.0)  1.9 (0.4-6.6) |
| High-lactose dairy  NIG  SCG | 1.4 (0-6.0)  1.4 (0-4.6) | 1.3 (0-4.3)  1.4 (0-3.6) | 1.3 (0-5.0)  1.5 (0-6.0) | 1.4 (0-5.4)  1.4 (0-6.0) | 1.4 (0-4.2)  1.4 (0-4.3) | 1.3 (0-5.4)  1.4 (0-5.3) | 1.2 (0-3.2)  1.3 (0-5.0) | 1.3 (0-6.0)  1.4 (0-5.0) |
| Low-lactose dairy  NIG  SCG | 0.3 (0-3.4)  0.3 (0-3.0) | 0.2 (0-1.3)  0.3 (0-1.9) | 0.3 (0-2.0)  0.2 (0-3.0) | 0.2 (0-1.3)  0.3 (0-2.0) | 0.2 (0-1.4)  0.3 (0-3.1) | 0.3 (0-2.0)  0.3 (0-3.1) | 0.3 (0-2.2)  0.3 (0-3.1) | 0.3 (0-4.1)  0.2 (0-1.4) |
| Users of LF/LR  NIG  SCG | 8  7 | 50  10 | 54  10 | 45  7 | 43  10 | 42  9 | 34  10 | 31  10 |

**Supplementary file B.** Median frequencies of intake (times per day) of the food categories, and the number of patients using lactose-free or lactose-reduced dairy products among patients with prostate cancer undergoing radiotherapy who received a nutrition intervention (NIG) and those who received standard care (SCG).

Abbreviations: LF, lactose-free; LR, lactose-reduced. Users of LR/LF indicates the number of patients who uses lactose-free or lactose-reduced dairy products. Note: Baseline, start of RT; 4 and 8 weeks, after RT onset; 2, 7, 12, 18 and 24 months, after RT completion.

**Supplementary file C.** Mean scores (SD) for the HRQOL domains assessing function and symptoms at baseline, the acute phase, and the late phase among patients with prostate cancer undergoing radiotherapy, who received a nutrition intervention (NIG) and those who received standard care (SCG).

|  | Baseline  Mean (SD) | | Acute phase^1^  Mean (SD) | | Late phase^2^  Mean (SD) | |
| --- | --- | --- | --- | --- | --- | --- |
|  | NIG | SCG | NIG | SCG | NIG | SCG |
|  | n =84 | n =85 | n =75 | n =82 | n =68 | n =82 |
| EORTC QLQ-C30 |  |  |  |  |  |  |
| Global health status | 75 (19) | 75 (18) | 67 (19) | 68 (19) | 74 (21) | 74 (18) |
| Physical functioning | 90 (16) | 93 (11) | 85 (16) | 88 (14) | 87 (15) | 88 (14) |
| Role functioning | 90 (18) | 90 (20) | 76 (24) | 78 (24) | 88 (18) | 88 (18) |
| Emotional functioning | 84 (18) | 85 (15) | 84 (14) | 83 (18) | 89 (13) | 86 (16) |
| Cognitive functioning | 90 (13) | 89 (16) | 88 (14) | 87 (15) | 89 (14) | 87 (15) |
| Social functioning | 86 (20) | 82 (20) | 76 (22) | 76 (22) | 81 (22) | 80 (22) |
| Fatigue | 19 (19) | 18 (21) | 31 (21) | 28 (19) | 24 (21) | 23 (20) |
| Nausea and vomiting | 1 (3) | 1 (5) | 5 (8) | 5 (9) | 2 (6) | 2 (5) |
| Pain | 12 (22) | 11 (19) | 17 (20) | 15 (20) | 13 (19) | 11 (18) |
| Dyspnoea | 16 (24) | 16 (20) | 21 (24) | 21 (19) | 22 (23) | 22 (19) |
| Insomnia | 27 (32) | 20 (27) | 32 (27) | 30 (27) | 24 (25) | 24 (24) |
| Appetite loss | 4 (14) | 2 (8) | 10 (14)* | 5 (11) | 5 (12) | 3 (8) |
| Financial difficulties | 5 (16) | 4 (14) | 6 (14) | 8 (17) | 3 (10) | 4 (14) |
| EORTC QLQ-PR25 |  |  |  |  |  |  |
| Sexual activity | 18 (22) | 18 (23) | 10 (16) | 10 (15) | 15 (21) | 17 (19) |
| Sexual functioning | 53 (24) | 48 (25) | 52 (20) | 50 (17) | 60 (17) | 53 (19) |
| Urinary symptoms | 21 (16) | 21 (18) | 39 (19) | 37 (17) | 22 (14) | 24 (16) |
| Hormonal treatment-related symptoms | 22 (14) | 22 (13) | 22 (13) | 25 (14) | 24 (15) | 26 (14) |

Scores ranges from 0-100 in EORTC QLQ-C30 and QLQ-PR25. * = significant association between the NI and loss of appetite, *p*=0.018.

^1^Acute phase: 4w, 8w and 2m

^2^Late phase: 7, 12, 18 and 24m.
